# 2-(Furan-2-yl)-3-hy­droxy-4*H*-chromen-4-one

**DOI:** 10.1107/S1600536810053596

**Published:** 2011-01-08

**Authors:** Michał Wera, Vasyl G. Pivovarenko, Artur Sikorski, Tadeusz Lis, Jerzy Błażejowski

**Affiliations:** a Faculty of Chemistry, University of Gdańsk, J. Sobieskiego 18, 80-952 Gdańsk, Poland; b Faculty of Chemistry, Kyiv Taras Shevchenko National University, Volodymyrska 64, 01033 Kyiv, Ukraine; c Faculty of Chemistry, University of Wrocław, F. Joliot-Curie 14, 50-383 Wrocław, Poland

## Abstract

In the crystal structure of the title compound, C_13_H_8_O_4_, the inversely oriented mol­ecules form inversion dimers through pairs of O—H⋯O hydrogen-bonding inter­actions. An intramolecular O—H⋯O hydrogen bond occurs. In the packing of the mol­ecules, the nearly planar 2-(furan-2-yl)-4*H*-chromene units [dihedral angle between the chromene and furan rings = 3.8 (1)°] are either parallel or inclined at an angle of 80.7 (1)°.

## Related literature

For general features of flavonols (derivatives of 3-hy­droxy-2-phenyl-4*H*-chromen-4-one), see: Klymchenko *et al.* (2003 ▶); Sengupta & Kasha (1979 ▶). For related structures, see: Etter *et al.* (1986 ▶); Waller *et al.* (2003 ▶). For inter­molecular inter­actions, see: Aakeröy *et al.* (1992 ▶); Novoa *et al.* (2006 ▶). For the synthesis, see: Klymchenko *et al.* (2003 ▶).
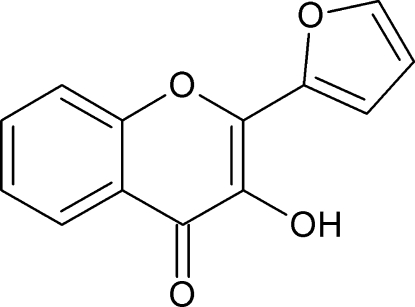


## Experimental

### 

#### Crystal data

C_13_H_8_O_4_
*M* *_r_* = 228.19
Monoclinic, 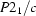

*a* = 14.365 (8) Å
*b* = 4.421 (3) Å
*c* = 17.086 (10) Å
β = 110.91 (5)°
*V* = 1013.6 (11) Å^3^
*Z* = 4
Mo *K*α radiation
μ = 0.11 mm^−1^
*T* = 100 K
0.40 × 0.40 × 0.14 mm

#### Data collection

Kuma KM4 CCD κ-geometry diffractometer
7132 measured reflections
1779 independent reflections
1436 reflections with *I* > 2σ(*I*)
*R*_int_ = 0.030

#### Refinement

*R*[*F* ^2^ > 2σ(*F* ^2^)] = 0.037
*wR*(*F* ^2^) = 0.092
*S* = 1.10
1779 reflections
158 parameters
H atoms treated by a mixture of independent and constrained refinement
Δρ_max_ = 0.21 e Å^−3^
Δρ_min_ = −0.21 e Å^−3^

### 

Data collection: *CrysAlis CCD* (Oxford Diffraction, 2003 ▶); cell refinement: *CrysAlis RED* (Oxford Diffraction, 2003 ▶); data reduction: *CrysAlis RED*; program(s) used to solve structure: *SHELXS97* (Sheldrick, 2008 ▶); program(s) used to refine structure: *SHELXL97* (Sheldrick, 2008 ▶); molecular graphics: *ORTEP-3* (Farrugia, 1997 ▶); software used to prepare material for publication: *SHELXL97* and *PLATON* (Spek, 2009 ▶).

## Supplementary Material

Crystal structure: contains datablocks global, I. DOI: 10.1107/S1600536810053596/ng5090sup1.cif

Structure factors: contains datablocks I. DOI: 10.1107/S1600536810053596/ng5090Isup2.hkl

Additional supplementary materials:  crystallographic information; 3D view; checkCIF report

## Figures and Tables

**Table 1:** Hydrogen-bond geometry (Å, °)

*D*—H⋯*A*	*D*—H	H⋯*A*	*D*⋯*A*	*D*—H⋯*A*
C6—H6⋯O12^i^	0.97 (2)	2.53 (2)	3.352 (3)	142 (2)
O11—H11⋯O12	0.89 (2)	2.37 (2)	2.776 (3)	108 (2)
O11—H11⋯O12^ii^	0.89 (2)	1.87 (2)	2.683 (3)	152 (2)

Symmetry codes: (i) 
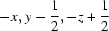
; (ii) 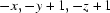.

**Fig. 1.**
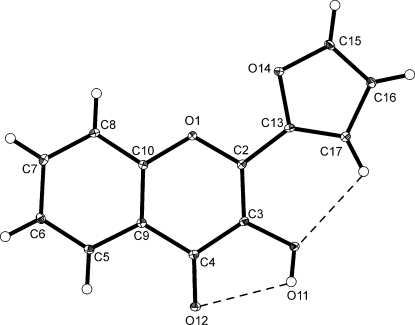
The molecular structure of the title compound showing the atom-labeling scheme. Displacement ellipsoids are drawn at the 25% probability level, and H atoms are shown as small spheres of arbitrary radius. The O–H···O and C–H···O hydrogen bonds are represented by dashed lines.

**Fig. 2.**
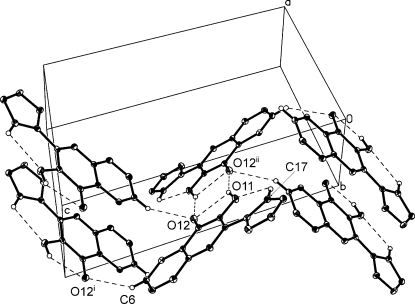
The arrangement of the molecules in the crystal structure. The O–H···O and C–H···O interactions are represented by dashed lines. H atoms not involved in interactions have been omitted. [Symmetry codes: (i) –x, y – 1/2, –z + 1/2; (ii) –x, –y + 1, –z + 1.]

## supplementary crystallographic information

### Comment

The structure of 2-(furan-2-yl)-3-hydroxy-4*H*-chromen-4-one is presented. This compound, in which Excited State Intramolecular Proton Transfer (ESIPT) takes place (Sengupta & Kasha, 1979), is a good candidate for a fluorescent probe sensitive to the properties of a medium (Klymchenko *et al.*, 2003).

In the title compound (Fig. 1), the bond lengths and angles characterizing the geometry of the 4*H*-chromen-4-one moiety are similar to those in 2-phenyl-4*H*-chromen-4-one (Waller *et al.*, 2003) and 3-hydroxy-2-phenyl-4*H*-chromen-4-one (Etter *et al.*, 1986). The average deviations from planarity of the phenyl, 4*H*-chromene and 2-(furan-2-yl)-4*H*-chromene cores are 0.0024 (2), 0.0046 (2) and 0.0298 (2), respectively, which implies that the molecule is practically planar (the dihedral angle between the planes of the 4*H*-chromene and furanyl fragments is only 3.8 (1)°). Intramolecular O–H···O and C–H···O interactions (Table 1, Figs. 1 and 2) undoubtedly make the molecule more rigid and contribute to its planarity, the former being the one involved in the ESIPT characteristic of flavonols (Sengupta & Kasha, 1979). The mean planes of adjacent 2-(furan-2-yl)-4*H*-chromene moieties are either parallel (remain at an angle 0.0 (1)°) in the crystal lattice or are inclined at an angle of 80.7 (1)°.

In the crystal structure, the inversely oriented molecules form dimers through a pair of O–H···O (Aakeröy *et al.*, 1992) interactions (Table 1, Fig. 2). Adjacent dimers are linked by C–H···O (Novoa *et al.*, 2006) interactions (Table 1, Fig. 2). The crystal structure is stabilized by these specific interactions, as well as by non-specific dispersive interactions.

### Experimental

The title compound was obtained by means of the oxidative heterocyclization of 3-(furan-2-yl)-1-(2-hydroxyphenyl)prop-2-en-1-one, synthesized by the condensation of 1-(2-hydroxyphenyl)ethanone with furan-2-carbaldehyde in methanol/50% aqueous NaOH (1/1 *v*/*v*), in alkaline methanol/H_2_O_2_ (Klymchenko *et al.*, 2003). The product was separated by filtration, and greenish-yellow crystals suitable for X-ray investigations were grown from ethanol (m.p. = 445 – 446 K).

### Refinement

H atoms involved in C–H···O and O–H···O interactions were located on a difference Fourier map and refined isotropically with *U*_iso_(H) = 1.2*U*_eq_(C) and *U*_iso_(H) = 1.5*U*_eq_(O), respectively. H atoms of other C–H bonds were positioned geometrically, with C–H = 0.95 Å, and constrained to ride on their parent atoms with *U*_iso_(H) = 1.2*U*_eq_(C).

### Crystal data

**Table:**

C_13_H_8_O_4_	*F*(000) = 472
*M**_r_* = 228.19	*D*_x_ = 1.495 Mg m^−^^3^
Monoclinic, *P*2_1_/*c*	Mo *K*α radiation, λ = 0.71073 Å
Hall symbol: -P 2ybc	Cell parameters from 1436 reflections
*a* = 14.365 (8) Å	θ = 3.4–25.0°
*b* = 4.421 (3) Å	µ = 0.11 mm^−^^1^
*c* = 17.086 (10) Å	*T* = 100 K
β = 110.91 (5)°	Plate, greenish-yellow
*V* = 1013.6 (11) Å^3^	0.40 × 0.40 × 0.14 mm
*Z* = 4	

### Data collection

**Table:**

Kuma KM4 CCD κ-geometry diffractometer	1436 reflections with *I* > 2σ(*I*)
Radiation source: fine-focus sealed tube	*R*_int_ = 0.030
graphite	θ_max_ = 25.0°, θ_min_ = 3.4°
ω scans	*h* = −17→17
7132 measured reflections	*k* = −4→5
1779 independent reflections	*l* = −20→18

### Refinement

**Table:**

Refinement on *F*^2^	Primary atom site location: structure-invariant direct methods
Least-squares matrix: full	Secondary atom site location: difference Fourier map
*R*[*F*^2^ > 2σ(*F*^2^)] = 0.037	Hydrogen site location: inferred from neighbouring sites
*wR*(*F*^2^) = 0.092	H atoms treated by a mixture of independent and constrained refinement
*S* = 1.10	*w* = 1/[σ^2^(*F*_o_^2^) + (0.0517*P*)^2^ + 0.0439*P*] where *P* = (*F*_o_^2^ + 2*F*_c_^2^)/3
1779 reflections	(Δ/σ)_max_ < 0.001
158 parameters	Δρ_max_ = 0.21 e Å^−^^3^
0 restraints	Δρ_min_ = −0.21 e Å^−^^3^

### Special details

**Table:**

Geometry. All e.s.d.'s (except the e.s.d. in the dihedral angle between two l.s. planes) are estimated using the full covariance matrix. The cell e.s.d.'s are taken into account individually in the estimation of e.s.d.'s in distances, angles and torsion angles; correlations between e.s.d.'s in cell parameters are only used when they are defined by crystal symmetry. An approximate (isotropic) treatment of cell e.s.d.'s is used for estimating e.s.d.'s involving l.s. planes.
Refinement. Refinement of *F*^2^ against ALL reflections. The weighted *R*-factor *wR* and goodness of fit *S* are based on *F*^2^, conventional *R*-factors *R* are based on *F*, with *F* set to zero for negative *F*^2^. The threshold expression of *F*^2^ > σ(*F*^2^) is used only for calculating *R*-factors(gt) *etc*. and is not relevant to the choice of reflections for refinement. *R*-factors based on *F*^2^ are statistically about twice as large as those based on *F*, and *R*- factors based on ALL data will be even larger.

### Fractional atomic coordinates and isotropic or equivalent isotropic displacement parameters (Å^2^)

**Table:**

	*x*	*y*	*z*	*U*_iso_*/*U*_eq_	
O1	0.30889 (8)	0.5549 (2)	0.43752 (6)	0.0209 (3)	
C2	0.27113 (12)	0.6670 (4)	0.49500 (9)	0.0189 (4)	
C3	0.17955 (12)	0.5867 (4)	0.49522 (9)	0.0192 (4)	
C4	0.11644 (12)	0.3802 (4)	0.43368 (9)	0.0200 (4)	
C5	0.10589 (12)	0.0603 (4)	0.30910 (9)	0.0212 (4)	
H5	0.0416	−0.0076	0.3051	0.025*	
C6	0.14678 (13)	−0.0412 (4)	0.25248 (10)	0.0232 (4)	
H6	0.1102 (13)	−0.177 (4)	0.2072 (11)	0.028*	
C7	0.24244 (13)	0.0564 (4)	0.25881 (10)	0.0248 (4)	
H7	0.2707	−0.0148	0.2197	0.030*	
C8	0.29541 (13)	0.2533 (4)	0.32077 (10)	0.0229 (4)	
H8	0.3601	0.3182	0.3250	0.027*	
C9	0.15832 (12)	0.2640 (4)	0.37305 (9)	0.0193 (4)	
C10	0.25266 (12)	0.3566 (4)	0.37755 (9)	0.0189 (4)	
O11	0.14832 (9)	0.7044 (3)	0.55453 (7)	0.0266 (3)	
H11	0.0840 (16)	0.665 (5)	0.5432 (12)	0.040*	
O12	0.03228 (8)	0.3089 (3)	0.43240 (7)	0.0277 (3)	
C13	0.33852 (12)	0.8757 (4)	0.55296 (9)	0.0198 (4)	
O14	0.42459 (8)	0.9419 (3)	0.53893 (7)	0.0245 (3)	
C15	0.47607 (12)	1.1419 (4)	0.59996 (10)	0.0264 (4)	
H15	0.5387	1.2273	0.6053	0.032*	
C16	0.42634 (12)	1.2018 (4)	0.65152 (10)	0.0243 (4)	
H16	0.4471	1.3323	0.6988	0.029*	
C17	0.33603 (13)	1.0302 (4)	0.62119 (10)	0.0224 (4)	
H17	0.2820 (13)	1.024 (4)	0.6427 (10)	0.027*	

### Atomic displacement parameters (Å^2^)

**Table:**

	*U*^11^	*U*^22^	*U*^33^	*U*^12^	*U*^13^	*U*^23^
O1	0.0202 (6)	0.0231 (6)	0.0215 (6)	−0.0020 (5)	0.0099 (5)	−0.0031 (5)
C2	0.0205 (9)	0.0197 (8)	0.0168 (8)	0.0034 (7)	0.0072 (7)	0.0038 (7)
C3	0.0188 (9)	0.0231 (9)	0.0162 (8)	0.0023 (7)	0.0069 (7)	0.0040 (7)
C4	0.0192 (9)	0.0211 (9)	0.0195 (8)	0.0019 (7)	0.0068 (7)	0.0053 (7)
C5	0.0186 (9)	0.0220 (9)	0.0212 (8)	0.0007 (7)	0.0050 (7)	0.0036 (7)
C6	0.0256 (10)	0.0219 (9)	0.0196 (9)	−0.0008 (7)	0.0049 (7)	0.0010 (7)
C7	0.0315 (10)	0.0231 (9)	0.0232 (9)	0.0034 (8)	0.0141 (8)	0.0014 (7)
C8	0.0208 (9)	0.0247 (9)	0.0257 (9)	−0.0013 (7)	0.0115 (7)	0.0014 (7)
C9	0.0198 (9)	0.0200 (9)	0.0170 (8)	0.0020 (7)	0.0051 (7)	0.0044 (6)
C10	0.0192 (8)	0.0180 (9)	0.0187 (8)	0.0010 (7)	0.0055 (7)	0.0028 (7)
O11	0.0201 (7)	0.0390 (8)	0.0226 (6)	−0.0047 (6)	0.0100 (5)	−0.0072 (5)
O12	0.0202 (7)	0.0377 (7)	0.0265 (6)	−0.0055 (5)	0.0099 (5)	−0.0040 (5)
C13	0.0164 (8)	0.0223 (9)	0.0207 (8)	0.0020 (7)	0.0066 (7)	0.0059 (7)
O14	0.0203 (6)	0.0280 (7)	0.0265 (6)	−0.0054 (5)	0.0100 (5)	−0.0047 (5)
C15	0.0201 (9)	0.0258 (10)	0.0290 (9)	−0.0055 (7)	0.0037 (7)	−0.0052 (8)
C16	0.0259 (9)	0.0228 (9)	0.0219 (8)	0.0000 (7)	0.0056 (7)	−0.0004 (7)
C17	0.0221 (9)	0.0238 (9)	0.0211 (8)	0.0022 (7)	0.0075 (7)	0.0021 (7)

### Geometric parameters (Å, °)

**Table:**

O1—C10	1.371 (2)	C7—H7	0.9500
O1—C2	1.3729 (19)	C8—C10	1.397 (2)
C2—C3	1.364 (2)	C8—H8	0.9500
C2—C13	1.444 (2)	C9—C10	1.391 (2)
C3—O11	1.3503 (19)	O11—H11	0.89 (2)
C3—C4	1.443 (2)	C13—C17	1.362 (2)
C4—O12	1.2419 (19)	C13—O14	1.372 (2)
C4—C9	1.464 (2)	O14—C15	1.366 (2)
C5—C6	1.374 (2)	C15—C16	1.344 (2)
C5—C9	1.409 (2)	C15—H15	0.9500
C5—H5	0.9500	C16—C17	1.431 (3)
C6—C7	1.407 (2)	C16—H16	0.9500
C6—H6	0.971 (18)	C17—H17	0.971 (17)
C7—C8	1.372 (2)		
			
C10—O1—C2	119.19 (13)	C10—C8—H8	120.6
C3—C2—O1	122.39 (15)	C10—C9—C5	118.13 (15)
C3—C2—C13	125.15 (14)	C10—C9—C4	119.72 (15)
O1—C2—C13	112.46 (14)	C5—C9—C4	122.15 (15)
O11—C3—C2	118.75 (15)	O1—C10—C9	122.13 (14)
O11—C3—C4	120.01 (14)	O1—C10—C8	116.11 (14)
C2—C3—C4	121.24 (14)	C9—C10—C8	121.76 (15)
O12—C4—C3	121.87 (15)	C3—O11—H11	110.9 (13)
O12—C4—C9	122.81 (15)	C17—C13—O14	110.06 (15)
C3—C4—C9	115.33 (14)	C17—C13—C2	133.72 (16)
C6—C5—C9	120.69 (16)	O14—C13—C2	116.21 (14)
C6—C5—H5	119.7	C15—O14—C13	106.30 (13)
C9—C5—H5	119.7	C16—C15—O14	111.01 (15)
C5—C6—C7	119.78 (16)	C16—C15—H15	124.5
C5—C6—H6	121.1 (10)	O14—C15—H15	124.5
C7—C6—H6	119.1 (10)	C15—C16—C17	106.47 (15)
C8—C7—C6	120.76 (15)	C15—C16—H16	126.8
C8—C7—H7	119.6	C17—C16—H16	126.8
C6—C7—H7	119.6	C13—C17—C16	106.17 (16)
C7—C8—C10	118.87 (16)	C13—C17—H17	125.5 (10)
C7—C8—H8	120.6	C16—C17—H17	128.4 (10)
			
C10—O1—C2—C3	0.5 (2)	C2—O1—C10—C9	−0.2 (2)
C10—O1—C2—C13	−179.14 (13)	C2—O1—C10—C8	179.56 (13)
O1—C2—C3—O11	179.20 (13)	C5—C9—C10—O1	179.80 (14)
C13—C2—C3—O11	−1.2 (2)	C4—C9—C10—O1	0.1 (2)
O1—C2—C3—C4	−0.8 (2)	C5—C9—C10—C8	0.1 (2)
C13—C2—C3—C4	178.82 (14)	C4—C9—C10—C8	−179.59 (14)
O11—C3—C4—O12	1.2 (2)	C7—C8—C10—O1	−179.42 (14)
C2—C3—C4—O12	−178.86 (15)	C7—C8—C10—C9	0.3 (2)
O11—C3—C4—C9	−179.30 (13)	C3—C2—C13—C17	3.7 (3)
C2—C3—C4—C9	0.7 (2)	O1—C2—C13—C17	−176.67 (16)
C9—C5—C6—C7	0.7 (2)	C3—C2—C13—O14	−175.90 (14)
C5—C6—C7—C8	−0.3 (2)	O1—C2—C13—O14	3.73 (19)
C6—C7—C8—C10	−0.2 (2)	C17—C13—O14—C15	−0.06 (17)
C6—C5—C9—C10	−0.6 (2)	C2—C13—O14—C15	179.64 (13)
C6—C5—C9—C4	179.08 (14)	C13—O14—C15—C16	0.35 (18)
O12—C4—C9—C10	179.17 (14)	O14—C15—C16—C17	−0.49 (19)
C3—C4—C9—C10	−0.4 (2)	O14—C13—C17—C16	−0.23 (18)
O12—C4—C9—C5	−0.5 (2)	C2—C13—C17—C16	−179.85 (17)
C3—C4—C9—C5	179.98 (14)	C15—C16—C17—C13	0.44 (19)

### Hydrogen-bond geometry (Å, °)

**Table:**

*D*—H···*A*	*D*—H	H···*A*	*D*···*A*	*D*—H···*A*
C6—H6···O12^i^	0.97 (2)	2.53 (2)	3.352 (3)	142 (2)
O11—H11···O12	0.89 (2)	2.37 (2)	2.776 (3)	108 (2)
O11—H11···O12^ii^	0.89 (2)	1.87 (2)	2.683 (3)	152 (2)
C17—H17···O11	0.97 (2)	2.43 (2)	2.907 (3)	110 (2)

Symmetry codes:  (i) −*x*, *y*−1/2, −*z*+1/2; (ii) −*x*, −*y*+1, −*z*+1.
